# Clinical and Laboratory Improvement in Hyperadrenergic Postural Orthostatic Tachycardia Syndrome (POTS) after COVID-19 Infection

**DOI:** 10.1155/2021/7809231

**Published:** 2021-08-18

**Authors:** Rebecca A. Ocher, Erika Padilla, Jonathan C. Hsu, Pam R. Taub

**Affiliations:** ^1^Department of Medicine, University of California, Los Angeles, Los Angeles, California, USA; ^2^Division of Cardiovascular Medicine, Department of Medicine, University of California, San Diego, California, USA

## Abstract

A 32-year-old woman with a history of symptomatic supraventricular tachycardia, inappropriate sinus tachycardia, and hyperadrenergic POTS was treated with ivabradine and metoprolol. She then presented with bradycardia and Mobitz II second-degree AV block on event monitoring six weeks after COVID-19 infection. Her post-viral workup revealed normalization of catecholamine levels and significant symptomatic improvement in heart rate. To the authors' knowledge, this is the first reported case of improvement in POTS after COVID-19 infection. As our understanding of COVID-19 continues to improve, it will be vital to better understand the impact of COVID-19 dysautonomia on cardiac patients.

## 1. Introduction

POTS is a multifactorial disorder that most commonly affects young women [1]. POTS is a form of dysautonomia, a term that encompasses abnormal function of the autonomic nervous system [2]. Dysautonomia can more specifically be defined as either an overactivity or a failure of the sympathetic or parasympathetic components of the autonomic nervous system [3]. The pathophysiology of POTS is complex and variable, with five primary subtypes of disease (neuropathic, hypovolemic, primary hyperadrenergic, joint hypermobility-related, and immune-related) [1]. The presentations of POTS are heterogeneous, and, although the patient described was diagnosed with hyperadrenergic POTS, the pathophysiology of her POTS was most likely multifactorial [4]. The precise etiology of POTS is unknown, but there are several known stressors that may precipitate POTS—viral infection, pregnancy, vaccination, trauma, and psychosocial stress [1]. It is not surprising, therefore, that many cases of post-COVID-19 POTS/dysautonomia have been reported [5]. As our understanding of COVID-19 continues to grow, it will be important to assess how this viral infection can affect both the incidence of POTS and the impact of viral infection on patients previously diagnosed with POTS.

## 2. Case Presentation

A 32-year-old woman with history of Hashimoto's thyroiditis and migraines presented in September 2019 with complaints of elevated heart rate. An episode of supraventricular tachycardia was captured on electrocardiogram, and she was referred to electrophysiology for ablation. She underwent a slow pathway ablation for AV node reentrant tachycardia in January 2020. Post ablation, there were no inducible arrhythmias. However, post ablation, she complained of elevated heart rate into the 150s. In April 2020, she underwent slow pathway ablation once more and an implantable cardiac monitor was placed. This implanted monitor revealed inappropriate sinus tachycardia, for which she was symptomatic. There was no workup done to assess for POTS. She was then prescribed ivabradine for inappropriate sinus tachycardia, which improved her symptoms. Unfortunately, she also experienced blurred vision associated with ivabradine, a side effect that she could not tolerate due to her work demands as an airfield controller.

In July 2020, she underwent a partial sinus node ablation, after her two slow pathway ablations. She remained symptomatic with an elevated heart rate. It was then recommended by her electrophysiologist that she undergo a complete sinus node ablation with pacemaker placement due to the nature of her vocation (which prohibits her from taking medications that could impair her performance).

She sought a second opinion from a general cardiologist on September 21, 2020. On evaluation, the increase in her heart rate from 108 bpm while supine to 125 bpm while standing was suggestive of a POTS diagnosis [1]. Furthermore, her supine norepinephrine was 677 pg/mL and her standing norepinephrine level was 855 pg/mL, suggestive of hyperadrenergic POTS (Table 1). After shared decision making, the patient elected to maximize medical management once more with metoprolol and ivabradine, as well as a supervised exercise program. She took a leave of absence from her work while on these medications and was monitored via a non-real-time heart monitor for two weeks.

**Figure 1:**
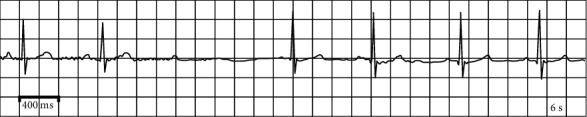
Zio XT Final Report 10/06/2020-10/19/2020 demonstrating Mobitz II second-degree AV block. 92 episodes of Mobitz II second-degree AV block were recorded.

Of note, the patient tested positive for SARS-CoV-2 by PCR on September 24, 2020. She wore her heart monitor from October 6 through October 19, 2020. The results of her non-real-time heart monitor became available on November 10, 2020, and demonstrated bradycardia as low as 31 beats per minute and Mobitz II second-degree AV block (Figure 1). Given this reading, she was advised to present to the emergency department, and she was subsequently admitted to the cardiology service.

At the time of admission, the patient reported poor compliance with ivabradine and metoprolol, only taking these medications occasionally in the four weeks prior to her admission. Her event monitor reported 92 episodes of Mobitz II second-degree AV block lasting a total of 6 minutes and 32 seconds over the course of two weeks (Figure 1).

During her admission, the patient was evaluated by the electrophysiology service and was noted to have dropped beats on telemetry but was asymptomatic during these. Because she had two slow pathway AVNRT ablations, her fast pathway likely had a longer refractory period and the dropped beats were likely secondary to transient AV block. Because she had normal PR and QRS intervals, malignant AV nodal and infranodal pathology was thought to be unlikely. Pacemaker placement was deemed unnecessary. Metoprolol and ivabradine were not felt to be the cause of her bradycardia as she reported poor compliance with these medications prior to her admission.

After discharge, she was seen in the outpatient cardiology clinic on November 13, 2020, and her POTS was reevaluated. Her heart rate was 86 bpm while supine and 97 bpm standing. Further, her supine and standing norepinephrine levels had markedly decreased to 203 pg/mL and 419 pg/mL, respectively. The patient also reported subjectively feeling better since discharge. Heart monitoring in November 2020 recorded mostly sinus rhythm, with occasional periods of Type II AV Block, both Mobitz I and Mobitz II second-degree AV block. Since discharge, the patient has remained stable from a cardiac standpoint without requiring any chronotropic medications.

## 3. Discussion

While there have been reported cases of reversible POTS [6], to the authors' knowledge, this is the first reported case of both clinical and laboratory improvement in a patient with POTS after COVID-19 infection. It is important to note that there has been a broad range of COVID-19 dysautonomia reported in the literature, ranging from labile pressor requirements in the ICU to dizziness and palpitations after discharge [3, 7, 8]. The precise pathophysiology of this postviral dysautonomia has not been identified to date, but both the cytokine storm and an autoimmune response have been postulated as possible mechanisms of this autonomic dysfunction [3, 7]. Even patients that experience a mild form of COVID-19 infection may be susceptible to the long-term effects of COVID-19 dysautonomia [9]. Additionally, sympathetic overactivation may play an important role in the pathophysiology of COVID-19 infection, becoming an important variable in baseline hyperadrenergic patients [10]. All of these factors may contribute to long-term sympathetic dysregulation and immune dysregulation in recovered patients.

In this patient, we postulate that the COVID-19 infection could have “reset” her sympathetic nervous system. To this end, her previously diagnosed POTS clinically improved. Many patients anecdotally have reported both improvements and flares of POTS with viral infection. It will be important to follow this patient over time and monitor symptoms, vitals, and laboratory values, as there is no data to suggest how long these postviral effects may last. As we continue to better understand COVID-19 dysautonomia, it will be important to assess the impact on patients who may develop POTS, as well as patients with previously diagnosed POTS. Regardless of the etiology of POTS, it is important to educate patients that this is a dynamic disease and may evolve over time and that infections can significantly impact the disease trajectory.

## Figures and Tables

**Table 1:** Supine and standing vital signs and norepinephrine levels before and after COVID-19 infection.

	Before COVID-19 infection 9/21/2020	After COVID-19 infection 11/13/2020
Blood pressure: supine	118/84 mmHg	122/75 mmHg
Blood pressure: standing	127/84 mmHg	122/88 mmHg
Heart rate: supine	108 bpm	86 bpm
Heart rate: standing	125 bpm	97 bpm
Norepinephrine: supine	677 pg/mL	203 pg/mL
Norepinephrine: standing	855 pg/mL	419 pg/mL

## Data Availability

Background information is available on the medical database PubMed.
